# TEAD4 Promotes Myogenic Differentiation of Porcine Skeletal Muscle Satellite Cells

**DOI:** 10.3390/ani16101546

**Published:** 2026-05-18

**Authors:** Huanhuan Zhou, Jiayi Zeng, Xiaoyu Zhang, Xinqi Zeng, Ke Xu, Hongbo Chen

**Affiliations:** Laboratory of Genetic Breeding, Reproduction and Precision Livestock Farming & Hubei Provincial Center of Technology Innovation for Domestic Animal Breeding, School of Animal Science and Nutritional Engineering, Wuhan Polytechnic University, Wuhan 430023, China; hhzhou@whpu.edu.cn (H.Z.); 18674210615@163.com (J.Z.); zhangxiaoyu1823@163.com (X.Z.); zxq07070116@163.com (X.Z.); 22113033@whpu.edu.cn (K.X.)

**Keywords:** *TEAD4*, porcine skeletal muscle satellite cells, myogenic differentiation, transcriptomics, AMPK signaling pathway

## Abstract

Skeletal muscle satellite cells are indispensable for muscle growth and regeneration, and their myogenic differentiation is precisely controlled by transcription factors. As a core member of the TEAD family, *TEAD4* participates in various biological processes, yet its function and regulatory mechanism in porcine skeletal muscle satellite cells (PSCs) remain largely unknown. High-purity PSCs were isolated and identified from 7-day-old Large White piglets. Combined approaches of siRNA-mediated *TEAD4* knockdown, RT-qPCR, Western blotting, immunofluorescence, EdU assays, and transcriptome sequencing were applied to explore the role of *TEAD4* during myogenic differentiation. *TEAD4* expression was gradually upregulated during PSC differentiation and positively correlated with myogenic marker genes. Knockdown of *TEAD4* did not affect PSC proliferation but significantly suppressed myogenic differentiation, as indicated by reduced expression of myogenic genes and blocked myotube formation. Transcriptomic analysis demonstrated that DEGs were highly enriched in metabolic pathways, particularly the AMPK signaling pathway. *TEAD4* knockdown led to excessive upregulation of *PRKAG3* and prominent induction of *SLC2A4*. Collectively, these results indicate that *TEAD4* promotes myogenic differentiation in PSCs, likely by maintaining metabolic homeostasis. This study provides the first characterization of *TEAD4* in porcine skeletal muscle satellite cells and demonstrates that it promotes myogenic differentiation.

## Simple Summary

Skeletal muscle satellite cells are essential for muscle growth and repair in pigs, and their differentiation is precisely regulated by multiple factors. *TEAD4* is an important regulator involved in various biological processes, but its specific role in porcine muscle development remains unclear. In this study, high-purity skeletal muscle satellite cells were isolated from 7-day-old Large White piglets, and the function of *TEAD4* was investigated using RNA interference. The results showed that *TEAD4* expression was significantly increased during myogenic differentiation. Knockdown of *TEAD4* did not affect cell proliferation but significantly inhibited myotube formation, which was accompanied by downregulated expression of myogenic genes and a decreased number of multinucleated myotubes. Transcriptomic analysis further revealed that *TEAD4* does not directly activate muscle-specific genes, but its knockdown leads to the enrichment of metabolic pathways, particularly the AMPK signaling pathway. This study shows that *TEAD4* promotes myogenic differentiation in porcine satellite cells.

## 1. Introduction

Skeletal muscle is the most abundant tissue in mammals and plays an irreplaceable role in maintaining structural integrity, supporting motor function, and regulating energy metabolism [1,2]. Its development and regeneration rely on satellite cells, a population of adult stem cells located between the basement membrane and sarcolemma of muscle fibers [3]. As the core effector cells for skeletal muscle growth, injury repair, and regeneration, satellite cells possess strong self-renewal and multi-directional differentiation potential [4,5,6,7,8,9]. PSCs have become an important model for studying muscle developmental mechanisms and livestock breeding [10].

*TEAD4* is a key member of the TEAD transcription factor family (*TEAD*1–4) and a core downstream effector of the Hippo signaling pathway [4]. In skeletal muscle, *TEAD4* is activated early in development and serves as a critical regulator during myogenic differentiation and myotube maturation [11,12]. It interacts with co-activators YAP/TAZ to modulate the expression or activity of core myogenic transcription factors such as MyoD and MyoG, thereby coordinating the proliferation–differentiation balance of myogenic cells [12]. Recent studies have demonstrated that *TEAD4* forms a transcriptional complex with MyoG and Klf5 to drive muscle-specific gene expression [11] and regulates insulin sensitivity through the TAZ-TEAD axis [13]. In mice, *TEAD4* has also been shown to directly activate the expression of myogenin and other differentiation-related genes during myogenesis [14].

Notably, the function of *TEAD4* in muscle development exhibits marked species-specificity. In mice, *TEAD4* promotes myogenic differentiation by directly activating the myogenic transcriptional network [14], and deletion of its co-activator VGLL3 leads to muscle fiber atrophy [15]. In contrast, *TEAD4* knockdown in bovine skeletal muscle satellite cells exerts little effect on myotube formation, suggesting a marginal role in cattle [16]. These contrasting observations suggest that the contribution of *TEAD4* to myogenic differentiation may vary across species. Furthermore, cis-regulatory element landscapes differ substantially between pig and mouse genomes [17], implying that the regulatory context for transcription factors may not be identical across species. Therefore, direct investigation of *TEAD4* in porcine satellite cells is necessary. In addition, in porcine muscle development, *TEAD4* has been implicated in fatty acid metabolism and muscle growth regulation [18,19]. However, to date, the function and regulatory mechanism of *TEAD4* in porcine skeletal muscle satellite cells remain largely unexplored.

Thus, this study employed high-purity porcine skeletal muscle satellite cells as a model to investigate the biological function and regulatory mechanism of *TEAD4* during myogenic differentiation. Through gene knockdown, transcriptome sequencing, and functional validation, this study provides the first characterization of *TEAD4* in porcine satellite cells.

## 2. Materials and Methods

### 2.1. Animals

One-week-old, healthy Large White piglets were obtained from a commercial pig farm in Wuhan, Hubei Province, China. Piglets were housed with their sows under standard commercial conditions. All animal care and experimental procedures were performed in accordance with the guidelines of the National Research Council’s Guide for the Care and Use of Laboratory Animals. The experimental protocol was approved by the Animal Ethics Committee of Wuhan Polytechnic University (Approval No. WPU202402001). Throughout the experiments, animal welfare principles were strictly followed. Procedures were optimized, and stress stimuli were minimized to reduce discomfort and pain, ensuring full compliance with ethical standards. No adverse events were observed during the study. Humane endpoints were not established as animals were euthanized prior to cell isolation, and no disease or pain models were used.

### 2.2. Cell Isolation, Culture, and Myogenic Differentiation

PSCs were isolated from the hindlimb muscles of 7-day-old Large White piglets after euthanasia. Muscle tissues were washed with PBS containing 1% antibiotic-antimycotic (AA; Life Technologies, Carlsbad, CA, USA, 15240-096), minced, and digested with 2.5 mg/mL collagenase type II (Life Technologies, 17101015) in a 37 °C water bath shaker for 2.5 h. This method was optimized in preliminary experiments to minimize cell damage and maximize cell viability. Digestion was terminated by adding DMEM (Hyclone, Logan, UT, USA, SH30022.01B) supplemented with 10% fetal bovine serum (FBS; Gibco, Grand Island, NY, USA, 10099141C). The cell suspension was sequentially filtered through 100-, 200-, and 400-mesh sieves to obtain a crude cell population. The filtered cells were washed with PBS and RPMI-1640 (Gibco, A10491-01) and then resuspended in growth medium consisting of RPMI-1640 supplemented with 20% FBS, 0.5% chicken embryo extract (CEE; Gemini, New York, NY, USA, 100-163P), 1% GlutaMax (Gibco, 35050-061), 1% non-essential amino acids (NEAA; Gibco, 11140-050), 1% AA, 1% gentamicin (Biosharp, Hefei, China, BS143-1g), and 2.5 μg/L basic fibroblast growth factor (bFGF; Gibco, 13256-029). To enrich satellite cells, differential plating was performed. The cell suspension was incubated in an uncoated dish for 2.5 h to remove rapidly adhering fibroblasts. Non-adherent cells were collected and transferred to Matrigel-coated plates (BD Biosciences, San Jose, CA, USA, 356234) for proliferation, with medium replaced every 24 h. When cells reached 80% confluence, differentiation was induced by switching to differentiation medium (DMEM containing 5% horse serum (HS; Hyclone, SH300074.02) and 1% AA).

### 2.3. siRNA Synthesis and Transfection

Three pairs of siRNAs targeting porcine *TEAD4* mRNA (NCBI Reference Sequence: NM_001142666.2) were designed and synthesized by Guangzhou RiboBio Co., Ltd. (Guangzhou, China) and designated as si-1, si-2, and si-3. All designed siRNA sequences were verified by NCBI BLAST online service https://blast.ncbi.nlm.nih.gov (accessed on 12 March 2024) to ensure target specificity and reduce potential off-target effects. Specific primers for *TEAD1*, *TEAD2*, and *TEAD3* were designed based on sequence homology to enable the specific detection of homologous genes. Transfection was performed in 12-well plates using the riboFECT™ CP Transfection Kit (RiboBio, China) according to the manufacturer’s instructions. A universal negative control and FAM/Cy5-labeled fluorescent control siRNA were used to evaluate transfection efficiency. Preliminary concentration tests were carried out to assess transfection performance and myogenic differentiation, and appropriate siRNA concentrations were selected for subsequent experiments. Knockdown efficiency was validated 24 h post-transfection. For differentiation assays, cells were induced to differentiate 24 h after transfection and cultured in differentiation medium for the indicated times. The siRNA sequences are listed in Table S1.

### 2.4. Quantitative Real-Time PCR (RT-qPCR)

Total RNA was extracted from PSCs using TRIzol reagent (Invitrogen, 15596-026, Waltham, MA, USA). Reverse transcription was performed using the PrimeScript RT Reagent Kit with gDNA Eraser (Perfect Real Time; TaKaRa, RR037A, Kyoto, Japan). RT-qPCR was carried out with TB Green^®^ Premix Ex Taq™ II (TaKaRa, RR820A, Kyoto, Japan) on a QuantStudio™ 1 Plus Real-Time PCR System (Thermo Fisher Scientific, Waltham, MA, USA). *GAPDH* was used as an internal control to normalize gene expression. Three biological replicates and three technical replicates were performed for each reaction. Relative mRNA expression levels were calculated using the 2^−ΔΔCt^ method. Primer sequences are listed in Table S2. Furthermore, all RT-qPCR amplified products were purified and subjected to Sanger sequencing to verify amplification specificity.

### 2.5. Western Blotting

PSCs cultured in 12-well plates were lysed on ice for 5 min using RIPA lysis buffer (Beyotime, Shanghai, China) containing PMSF. Protein lysates were collected, and total protein concentration was determined by BCA assay. Samples were adjusted to equal concentrations using RIPA lysis buffer and SDS-PAGE loading buffer (ABclonal, Wuhan, China). After vortexing and brief centrifugation, the samples were denatured by heating, separated by SDS-PAGE, and transferred onto PVDF membranes. The membranes were blocked with 5% non-fat milk in TBST for 2 h at room temperature on a shaker, followed by overnight incubation with primary antibodies at 4 °C. After three washes with TBST, the membranes were incubated with specific secondary antibodies for 1 h at room temperature in the dark. Protein bands were visualized using ECL reagent (Beyotime, China) and a Monad QuickChemi 5200 imaging system. Band intensities were quantified using ImageJ software (v1.54a), with β-actin as the loading control. Details of the primary and secondary antibodies are listed in Table S3.

### 2.6. Immunofluorescence

PSCs cultured in 12-well plates were fixed with pre-chilled 4% paraformaldehyde for 15 min and washed three times with PBS. Cells were permeabilized with 0.3% Triton X-100 for 10 min and washed three times with PBS. After blocking with PBS containing 3% BSA, 0.3% Triton X-100, and 10% FBS for 2 h, cells were incubated with primary antibodies overnight at 4 °C. The next day, cells were washed three times with PBST (PBS containing 0.1% Tween-20) and incubated with fluorophore-conjugated secondary antibodies for 1 h at room temperature in the dark. After PBST washing, nuclei were counterstained with DAPI (1:1000) for 10 min, washed, and examined under a microscope. Images were acquired using a microscope imaging system (REV32044; Thermo Fisher Scientific). Antibody details are listed in Table S3.

### 2.7. EdU Proliferation Assay

PSCs seeded in 12-well plates were cultured until 60–70% confluence was reached. The EdU working solution was prepared according to the manufacturer’s instructions of the BeyoClick™ EdU-555 Cell Proliferation Kit (Beyotime, China), and cells were incubated for 2 h. Following the kit protocol, cells were then fixed, permeabilized, and subjected to the Click reaction. After staining, images were acquired using a microscope imaging system. The numbers of EdU-positive cells and total cells in each field were recorded, and the percentage of proliferating cells was calculated as (EdU-positive cells/total cells) × 100%.

### 2.8. RNA Sequencing and Transcriptomic Analysis

Total RNA was extracted from PSCs using TRIzol reagent (TIANGEN, Beijing, China). RNA concentration and purity were determined using a NanoDrop 2000 spectrophotometer (Thermo Fisher Scientific, USA). Sequencing libraries were constructed using the VAHTS Universal V8 RNA-seq Library Prep Kit for MGI (Vazyme, Nanjing, China) according to the manufacturer’s instructions and sequenced on an Illumina NovaSeq 6000 platform (San Diego, CA, USA). Fastp (v0.23.2) was employed for quality control on the sequencing data, and quality assessment was visualized with FastQC. Adapters and low-quality reads were removed using Cutadapt to obtain high-quality clean data. The reference genome and annotation files were downloaded from a genome database. HISAT2 (v2.2.1) was used to build the genome index and align the clean reads to the reference genome. DESeq2 (v1.36.0) was employed to normalize the gene expression matrix and perform differential expression analysis. Differentially expressed genes (DEGs) were identified with the threshold of |log_2_(Fold Change)| ≥ 1 and *p*-value < 0.05. GO functional annotation and KEGG pathway enrichment analyses were conducted using the clusterProfiler package in R (v4.3.1), with significantly enriched terms defined by an adjusted *p*-value < 0.05. The results were visualized using the online data analysis and visualization platform https://www.bioinformatics.com.cn (accessed on 13 May 2026) [20].

### 2.9. Statistical Analysis

All data are presented as mean ± standard deviation (SD). Statistical analyses were performed using GraphPad Prism 10.6.0 (GraphPad Software, Boston, MA, USA). One-way or two-way analysis of variance (ANOVA), followed by Tukey’s post hoc test and Welch’s *t*-test, was used as appropriate. A *p*-value < 0.05 was considered statistically significant.

## 3. Results

### 3.1. Isolation and Identification of PSCs

**Figure 1:**
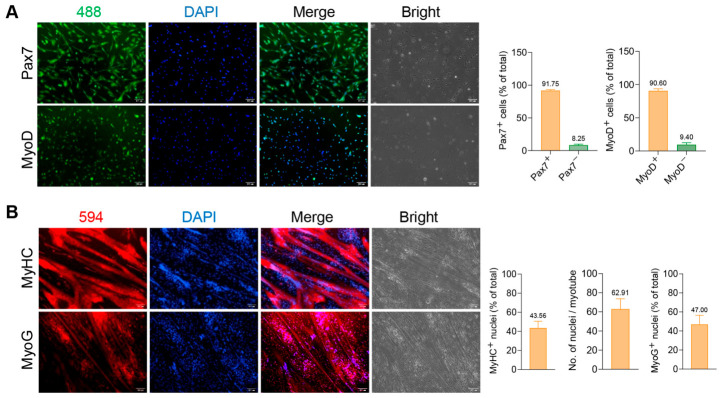
Isolation, purification, and myogenic differentiation potential of PSCs. (**A**) Immunofluorescence staining of Pax7 and MyoD in proliferative PSCs. Primary antibodies were detected with CoraLite^®^488-conjugated secondary antibodies (green), and nuclei were counterstained with DAPI (blue). Magnification: 100×; scale bar = 100 μm. Right panels: Quantitative analysis of Pax7^+^ (*n* = 4) and MyoD^+^ (*n* = 5) cell positivity rates, calculated as (number of positive cells/total DAPI-stained nuclei) × 100%. (**B**) Immunofluorescence staining of MyHC and MyoG in PSCs differentiated for 2 days. Primary antibodies were detected with CoraLite^®^594-conjugated secondary antibodies (red), and nuclei were counterstained with DAPI (blue). Magnification: 100×; scale bar = 100 μm. Right panels: quantitative analysis (*n* = 5). MyHC nuclei (%, Fusion index) = (Nuclei within MyHC^+^ myotubes/Total DAPI-stained nuclei) × 100, Nuclei per myotube = Nuclei within MyHC^+^ myotubes/Number of MyHC^+^ myotubes, MyoG^+^ nuclei (%) = (MyoG^+^ nuclei/Total DAPI-stained nuclei) × 100. Myotubes were defined as structures containing ≥2 nuclei.

PSCs were isolated from the hindlimb muscles of 7-day-old Large White piglets by enzymatic digestion followed by differential adhesion purification, and their biological characteristics and purity were systematically characterized. To assess the purity of the isolated PSCs, immunofluorescence staining was performed separately for Pax7 (a specific marker of satellite cells that labels both quiescent and proliferating cells [21,22]) and MyoD (a marker of myogenic commitment and early differentiation [23,24]) in proliferative cultures (Figure 1A). ImageJ-based quantitative analysis revealed that the proportion of Pax7^+^ cells was 91.75% and that of MyoD^+^ cells was 90.60% relative to the total number of DAPI-stained nuclei (Figure 1A). These high positivity rates confirmed that the isolated PSCs were of high purity.

Morphological changes during differentiation were observed under phase-contrast microscopy (Figure S2). At day 1 (D1), cells maintained a spindle-shaped morphology. By day 2 (D2), cells began to align and fuse, and at day 4 (D4), extensive multinucleated myotubes were formed, recapitulating the typical process of myogenic differentiation. The myogenic differentiation potential of PSCs was further validated by examining the expression of late and terminal myogenic markers in cultures differentiated for 2 days. Immunofluorescence staining was performed separately for MyHC (a terminal marker of myogenic differentiation [25,26] and MyoG (a late marker of myogenic differentiation [24,27]) (Figure 1B). Quantitative analysis showed that 43.56% of the total DAPI-stained nuclei were localized within MyHC^+^ myotubes, with an average of 62.91 nuclei per MyHC^+^ myotube, while 47.00% of the total DAPI-stained nuclei were MyoG^+^ (Figure 1B). Collectively, these results demonstrate that the isolated PSCs possess robust, directional myogenic differentiation potential, validating their suitability as a reliable cell model for subsequent functional investigations of *TEAD4*.

### 3.2. Expression Pattern of TEAD4 During PSC Myogenic Differentiation

**Figure 2:**
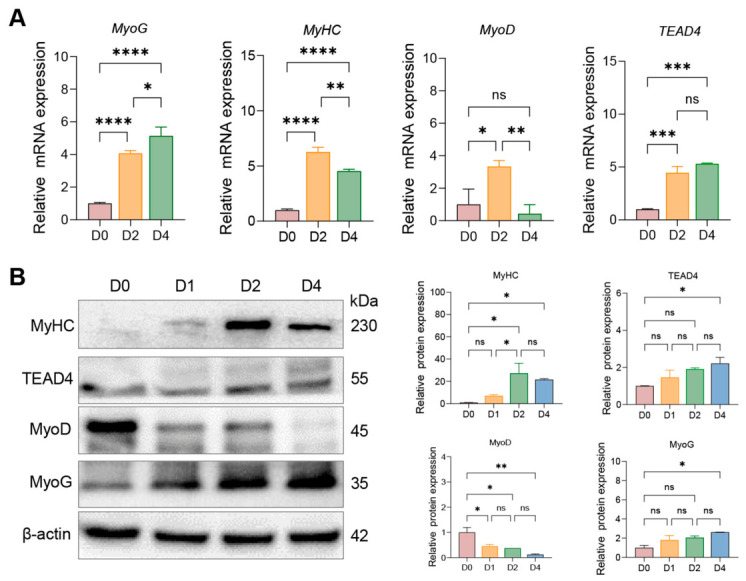
Expression dynamics of *TEAD4* and myogenic marker genes during PSC differentiation. (**A**) Relative mRNA expression levels of *MyoG*, *MyHC*, *MyoD*, and *TEAD4* at D0, D2, and D4, detected by RT-qPCR and normalized to *GAPDH*. *n* = 3. (**B**) Protein expression levels of MyHC, TEAD4, MyoD, and MyoG at D0, D1, D2, and D4, detected by Western blotting, with β-actin as loading control. Right panels: quantification of relative protein expression (normalized to β-actin, ImageJ). *n* = 2. Statistical significance was determined by one-way ANOVA followed by Tukey’s post hoc test. * *p* < 0.05, ** *p* < 0.01, *** *p* < 0.001, **** *p* < 0.0001; ns, *p* ≥ 0.05.

To investigate the role of *TEAD4* in myogenesis, the expression levels of *TEAD4* and key myogenic regulators (MyoG, MyHC, MyoD) during PSC differentiation were detected using RT-qPCR and Western blotting. RT-qPCR analysis revealed distinct temporal expression patterns (Figure 2A). The late myogenic markers *MyoG* and *MyHC* were progressively upregulated, with significant increases at D2 and further elevation at D4 compared to D0 (*p* < 0.0001). In contrast, the early marker *MyoD* showed a transient peak at D2 (*p* < 0.05 vs. D0), followed by a decline at D4. Notably, *TEAD4* mRNA was continuously and significantly upregulated throughout differentiation, mirroring the pattern of late myogenic markers (*p* < 0.001 at both D2 and D4 vs. D0).

Western blotting confirmed these trends at the protein level and revealed post-transcriptional regulation (Figure 2B). MyoG and MyHC proteins accumulated progressively, reaching significance at D4 (*p* < 0.05). TEAD4 protein showed a gradual increase, reaching statistical significance only at D4 (*p* < 0.05), indicating late-stage accumulation. Strikingly, MyoD protein decreased progressively from D1 onward (*p* < 0.05 at D1), contrasting with its mRNA pattern and suggesting post-translational degradation during differentiation. These results suggest that *TEAD4* expression correlates with terminal myogenic differentiation in PSCs, and the discordance between its mRNA and protein dynamics suggests potential post-transcriptional regulation.

### 3.3. Functional Role of TEAD4 in PSC Proliferation and Myogenic Differentiation

**Figure 3:**
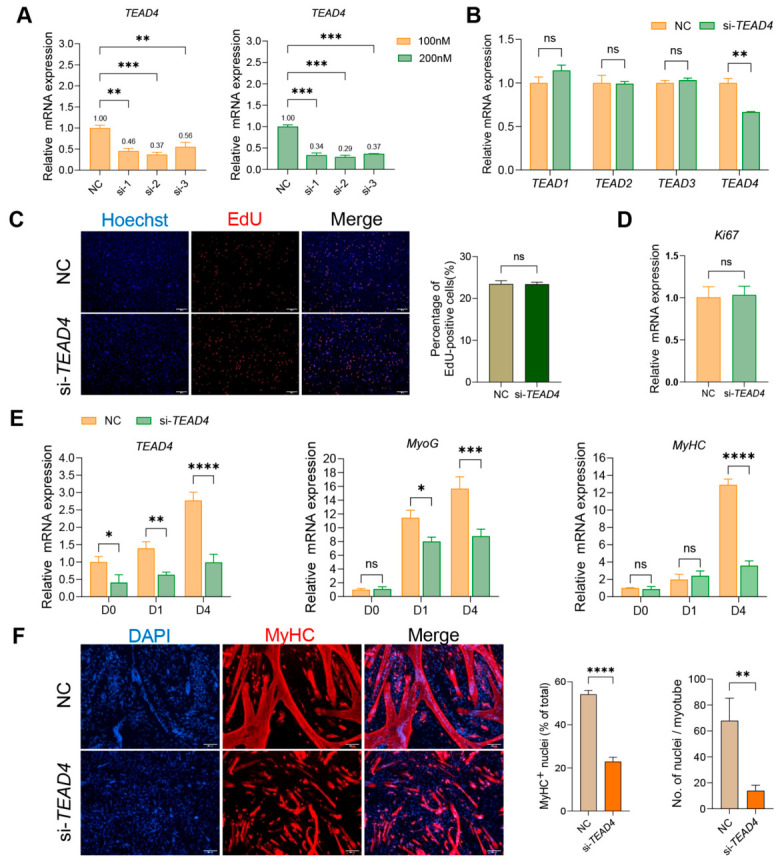
Effects of *TEAD4* knockdown on PSC proliferation and myogenic differentiation. (**A**) RT-qPCR analysis of *TEAD4* knockdown efficiency by three independent siRNA sequences at 100 nM and 200 nM. Expression normalized to *GAPDH*. *n* = 3. (**B**) mRNA expression of TEAD family genes (*TEAD*1–4) after *TEAD4* knockdown. *n* = 3. (**C**) EdU proliferation assay. EdU: red, proliferating cells; Hoechst: blue, nuclei. Magnification: 100×; scale bar = 100 μm. Right panel: quantification of EdU-positive cells (*n* = 8). (**D**) RT-qPCR analysis of *Ki67* mRNA expression normalized to *GAPDH* (*n* = 3). (**E**) Time-course RT-qPCR analysis of *TEAD4*, *MyoG*, and *MyHC* mRNA expression during differentiation at D0, D1, and D4 (*n* = 3). (**F**) Immunofluorescence staining of MyHC (red) and DAPI (blue) at day 3 of differentiation. Magnification: 100×; scale bar = 100 μm. Right panels: quantification of MyHC^+^ nuclei (% of total nuclei) and average nuclei per myotube (*n* = five fields). Statistical significance was determined by one- (**A**) or two-way ANOVA (**B**,**E**) with Tukey’s post hoc test or by Welch’s *t*-test (**C**,**D**,**F**). * *p* < 0.05, ** *p* < 0.01, *** *p* < 0.001, **** *p* < 0.0001; ns, *p* ≥ 0.05.

Three independent siRNA sequences targeting *TEAD4* were first evaluated. RT-qPCR analysis showed that all three sequences significantly reduced *TEAD4* mRNA expression at both 100 nM and 200 nM (*p* < 0.01, Figure 3A). The 200 nM si-2 (hereafter designated si-*TEAD4*) was selected for subsequent experiments due to its demonstrating the highest and most consistent knockdown efficiency. To rule out off-target effects or potential compensatory upregulation among TEAD family members, the mRNA expression of *TEAD1*, *TEAD2*, and *TEAD3* was detected following *TEAD4* knockdown. No significant changes were detected in *TEAD*1-3 expression (*p* > 0.05, Figure 3B), confirming the specificity of si-*TEAD4*.

The regulatory effect of *TEAD4* on PSC proliferation was subsequently assessed. EdU incorporation assays showed comparable percentages of EdU-positive cells between the si-*TEAD4* and negative control (NC) groups (*p* > 0.05, Figure 3C). Consistently, RT-qPCR analysis revealed no significant difference in *Ki67* mRNA levels (*p* > 0.05, Figure 3D). These results indicate that *TEAD4* does not influence PSC proliferation. In contrast, *TEAD4* knockdown markedly impaired myogenic differentiation. RT-qPCR analysis during differentiation revealed that *MyoG* mRNA levels were significantly reduced in si-*TEAD4* cells compared to NC groups at both D1 (*p* < 0.05) and D4 (*p* < 0.0001), whereas *MyHC* mRNA levels showed a significant reduction only at D4 (*p* < 0.0001, Figure 3E). Immunofluorescence staining for MyHC at day 3 of differentiation revealed markedly impaired myotube formation in si-*TEAD4* cultures (Figure 3F). Quantitative analysis confirmed a significant reduction in both the percentage of MyHC^+^ nuclei (*p* < 0.0001) and the average number of nuclei per myotube (*p* < 0.01), indicating that *TEAD4* knockdown impairs myotube maturation. Collectively, these results suggest that *TEAD4* acts as a positive regulator of PSC myogenic differentiation, with a critical role in terminal myotube formation, without affecting cell proliferation.

### 3.4. Effects of TEAD4 Knockdown on the Transcriptome of PSCs

**Figure 4:**
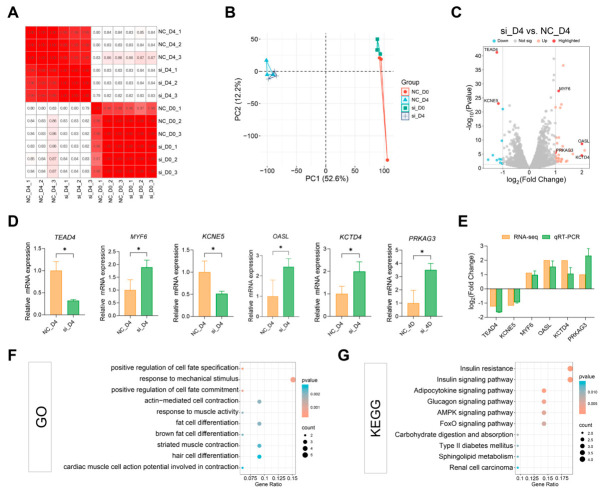
Transcriptomic profiling of *TEAD4*-knockdown PSCs during myogenic differentiation. (**A**) Pearson correlation heatmap of all samples. *n* = 3. (**B**) PCA plot of all samples. (**C**) Volcano plot of DEGs in the si_D4 vs. NC_D4 comparison. (**D**) Bar graphs showing the relative mRNA expression of representative DEGs in the NC_D4 and si_D4 groups, as detected by RT-qPCR. *n* = 3. Expression levels were normalized to *GAPDH* and are presented as fold change relative to the NC_D4 group. Statistical significance was determined by Welch’s *t*-test. * *p* < 0.05. (**E**) Scatter plot comparing log_2_(Fold Change) values from RNA-seq and RT-qPCR. (**F**) GO functional enrichment bubble plot of DEGs in the si_D4 vs. NC_D4 group. Terms were filtered with *p* < 0.05. (**G**) KEGG pathway enrichment bubble plot of DEGs in the si_D4 vs. NC_D4 group. Pathways were screened with *p* < 0.05.

To systematically analyze gene expression changes mediated by *TEAD4* knockdown, RNA-seq was performed on four cell populations at different differentiation stages, namely, NC_D0, NC_D4, si_D0 and si_D4. All samples generated high-quality clean data with Q20 > 99.34% and Q30 > 97.12% (Table S4). Pearson correlation analysis confirmed high consistency among biological replicates within each group (Figure 4A). PCA further revealed clear separation of samples according to differentiation stage, with the si_D4 group distinctly segregated from the NC_D4 group at the differentiation phase (Figure 4B). In the key comparison of si_D4 vs. NC_D4, 46 DEGs were identified, including 33 upregulated and 13 downregulated genes (Figure 4C and Figure S2A). The complete lists of DEGs for all four comparison groups are provided in Tables S5–S8. *TEAD4* expression was markedly downregulated in the si_D4 group, further verifying the stable and efficient siRNA-mediated knockdown. As expected, classic myogenic markers were upregulated during differentiation (Figure S2B). To validate the RNA-seq data, the expression of selected DEGs (*TEAD4*, *MYF6*, *KCNE5*, *OASL*, *KCTD4*, *PRKAG3*) was examined by RT-qPCR. The results showed high concordance with the RNA-seq data (Pearson’s *r* = 0.8653, *p* < 0.01; Figure 4D,E).

Functional enrichment analysis was performed to characterize the biological functions of DEGs in the si_D4 vs. NC_D4 comparison. GO analysis showed significant enrichment in biological processes related to muscle structure and contractile function, including actin-mediated cell contraction, striated muscle contraction, and response to mechanical stimulus (Figure 4F). KEGG pathway analysis revealed significant enrichment in the insulin signaling pathway, AMPK signaling pathway, and FoxO signaling pathway (Figure 4G). In contrast, no significant enrichment of core myogenic pathways (e.g., *MyoD*, *MyoG*, *Myf5*) was observed upon *TEAD4* knockdown. To further examine key metabolic genes from the RNA-seq data, the transcript levels of *PRKAG3* and *SLC2A4* (Figure S2C) were analyzed. *PRKAG3* expression increased by approximately 4.6-fold during normal myogenic differentiation and by approximately 11.6-fold upon *TEAD4* knockdown. In contrast, *SLC2A4* expression showed little change during normal differentiation but was markedly upregulated (31.2-fold) in the knockdown group.

## 4. Discussion

In this study, the biological function of *TEAD4* in the myogenic differentiation of PSCs was systematically investigated. The results suggest that *TEAD4* may exhibit species-specific regulatory characteristics in porcine muscle development, pointing to a potential role for *TEAD4* that warrants further investigation. *TEAD4* expression was continuously upregulated during PSC differentiation and positively correlated with late myogenic markers *MyoG* and *MyHC*. Functional assays confirmed that *TEAD4* knockdown had no effect on proliferation but significantly inhibited myotube formation, reduced the fusion index, and downregulated *MyoG* and *MyHC* expression. These results suggest that the functional role of *TEAD4* in pigs differs from its classical transcriptional activation role in mice [12] and its minimal role in cattle [16], aligning with previous observations linking *TEAD4* to fatty acid metabolism and muscle growth in pigs [18,19].

Transcriptome analysis revealed that *TEAD4* knockdown induced 46 DEGs significantly enriched in metabolic pathways, including the AMPK signaling pathway. Among these, *PRKAG3*, encoding the AMPKγ3 subunit, is specifically highly expressed in porcine muscle and plays a key role in energy metabolism and fiber-type composition [28,29]. Consistent with its role in differentiation-associated metabolic reprogramming, *PRKAG3* was significantly upregulated during normal myogenic differentiation. Notably, *TEAD4* knockdown led to an even more pronounced upregulation of *PRKAG3*. In contrast, *SLC2A4* expression remained unchanged during normal differentiation but was dramatically upregulated upon *TEAD4* knockdown [30]. These distinct expression patterns suggest that *TEAD4* may prevent excessive *PRKAG3* expression, while *SLC2A4* upregulation may represent a compensatory response to metabolic stress [30,31].

Compared with classical myogenic regulators such as Myomaker [32], which directly mediates myoblast fusion, and SMAD7 [9,33], which promotes myogenesis via epigenetic regulation and transcriptional coactivation, *TEAD4* appears to function as a metabolic modulator. Transcriptomic changes upon *TEAD4* knockdown were most prominent in metabolic pathways rather than core myogenic transcription factors, highlighting *TEAD4* as a unique regulator linking energy metabolism to differentiation. Mechanistically, excessive *PRKAG3* upregulation may influence AMPK signaling, which suppresses mTORC1 activity and protein synthesis when activated [34]. Notably, TAZ has been shown to interact with TEAD4 to induce *Irs1* expression and promote insulin-stimulated glucose uptake in muscle cells [13], further supporting the role of *TEAD4* in metabolic regulation. Consistent with this, *TEAD4* knockdown impaired myotube formation, suggesting that dysregulated AMPK signaling hinders the protein synthesis required for myotube maturation. Meanwhile, AMPK activation promotes GLUT4 translocation and glucose uptake [31], and the dramatic upregulation of *SLC2A4* may represent a compensatory response to maintain glucose homeostasis [30].

In the context of pig breeding, *PRKAG3* polymorphisms are closely associated with meat quality traits such as muscle glycogen content, meat color, and drip loss, making it a well-established candidate gene [35,36]. The excessive upregulation of *PRKAG3* upon *TEAD4* knockdown suggests a potential regulatory link between *TEAD4* and *PRKAG3*. Excessive *PRKAG3* upregulation may disturb AMPK signaling homeostasis and thus inhibit myotube maturation. From a theoretical perspective, *TEAD4* may serve as an upstream regulator of *PRKAG3* for future investigation, though its applicability as a marker-assisted breeding target requires further experimental evidence, including protein-level validation and in vivo functional studies [37,38]. In summary, this study provides preliminary evidence that *TEAD4* positively regulates myogenic differentiation in porcine satellite cells, likely through modulating energy metabolism-related pathways. These findings offer new insights into porcine muscle development and highlight *TEAD4* as a gene of interest for future studies on meat production traits. Due to the inherent limitations of primary porcine muscle cells, complementary overexpression and rescue experiments were not conducted. Moreover, the association between *TEAD4* and AMPK signaling is based solely on transcriptomic data and remains to be verified in further studies.

## 5. Conclusions

In this study, *TEAD4* is suggested to be a positive regulator of myogenic differentiation in porcine skeletal muscle satellite cells. *TEAD4* expression was progressively upregulated during differentiation and positively correlated with late myogenic markers, while *TEAD4* knockdown significantly impaired myotube formation without affecting cell proliferation. Transcriptome analysis revealed that *TEAD4* knockdown led to excessive upregulation of *PRKAG3* and dramatic upregulation of *SLC2A4*. Based on these findings, it is suggested that *TEAD4* may regulate metabolism-related pathways to promote myotube maturation, and its deficiency disrupts AMPK signaling balance, thereby impairing myotube maturation. This study provides new insights into the molecular mechanisms underlying porcine muscle development and suggests that *TEAD4* is a promising target for future mechanistic and in vivo studies.

## Data Availability

Data will be made available upon reasonable request.

## Supplementary Materials

The following supporting information can be downloaded at https://www.mdpi.com/article/10.3390/ani16101546/s1: Figure S1: Morphological changes in PSCs during differentiation. D1, D2, and D4 represent 1, 2, and 4 days of differentiation, respectively. Upper panel: 40× magnification, scale bar = 200 μm. Lower panel: 100× magnification, scale bar = 100 μm.; Figure S2: Summary of DEGs and changes in expression of key metabolic genes. (A) Number of up- and downregulated DEGs in each comparison group. (B) Volcano plots of DEGs in the NC_D4 vs. NC_D0 and si_D4 vs. si_D0 comparisons. (C) Changes in expression of *PRKAG3* and *SLC2A4* under different conditions. Bar graphs show fold change in four comparison groups.; Table S1: siRNA sequences.; Table S2: Primers used for RT-qPCR.; Table S3: List of primary and secondary antibodies used in this study.; Table S4: Quality control and mapping results of transcriptome sequencing data.; Table S5: DEGs in NC_D4 vs. NC_D0.; Table S6: DEGs in si_D4 vs. si_D0.; Table S7: DEGs in NC_D0 vs. si_D0.; Table S8: DEGs in si_D4 vs. NC_D4.
