# Supplementary figures and images for: TEAD4 Promotes Myogenic Differentiation of Porcine Skeletal Muscle Satellite Cells
Source: Animals (Basel). 2026 May 18;16(10):1546. doi: 10.3390/ani16101546 (PMC13203076; doi:10.3390/ani16101546)

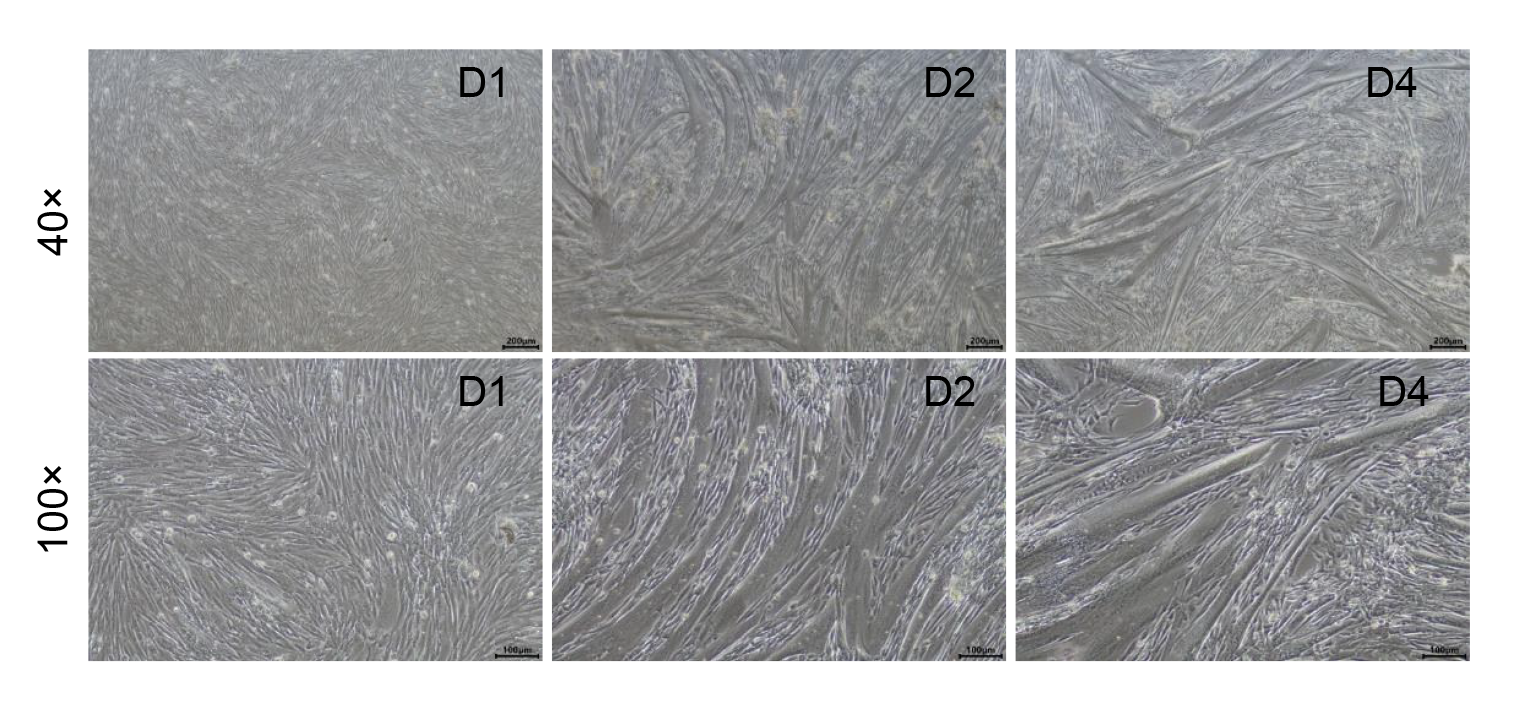

Supplement: Supplementary file 1 [file animals-16-01546-s001.zip › Fig.S1.png]

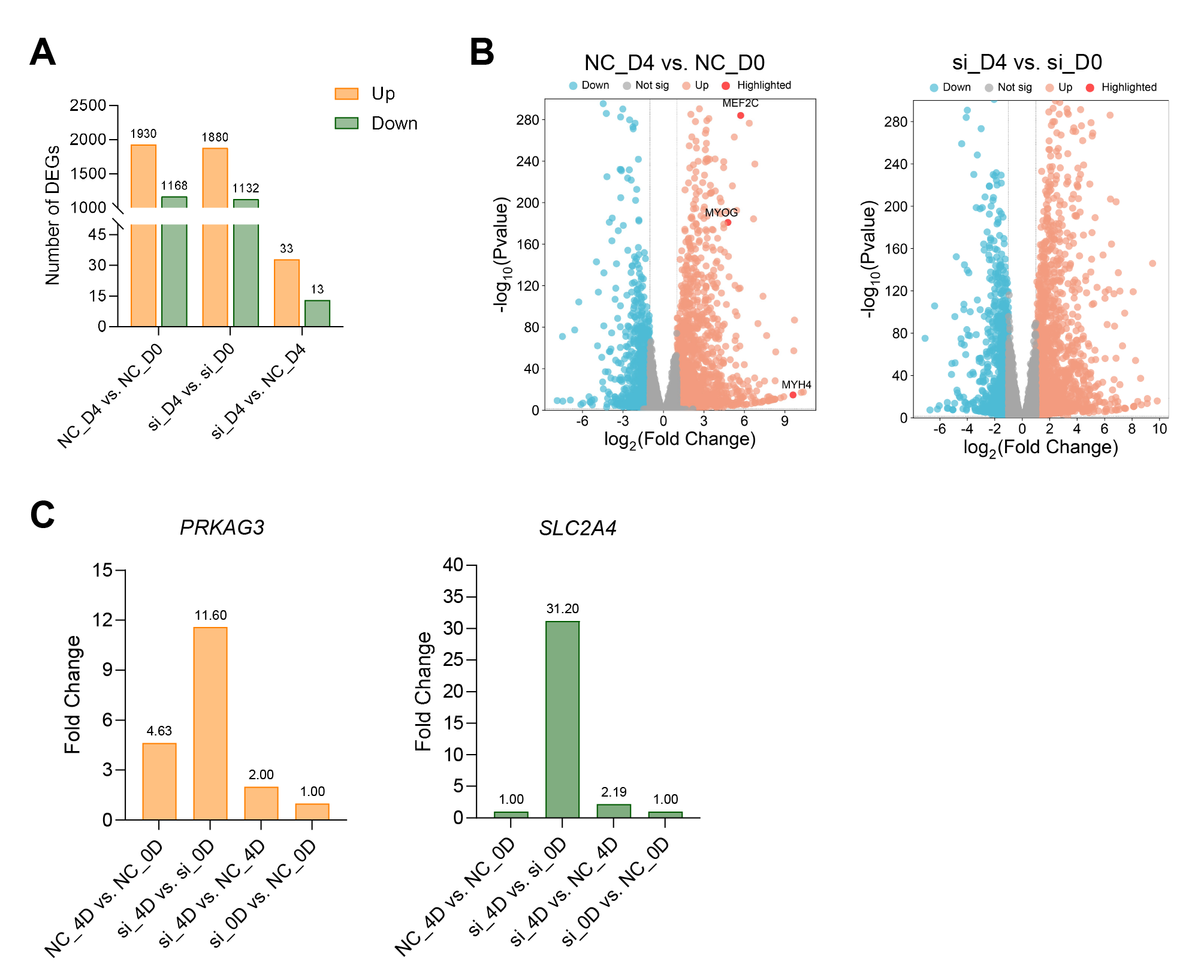

Supplement: Supplementary file 1 [file animals-16-01546-s001.zip › Fig.S2.png]
